# 18:0 Lyso PC Derived by Bioactivity-Based Molecular Networking from Lentil Mutant Lines and Its Effects on High-Fat Diet-Induced Obese Mice

**DOI:** 10.3390/molecules26247547

**Published:** 2021-12-13

**Authors:** Ah-Reum Han, Hae Ran Park, Geum Jin Kim, Bo-Ram Kim, Ye-Ram Kim, Hyeon Hwa Park, Jisu Park, Chang Hyun Jin, Jung Min Kim, Soon-Jae Kwon, Jin-Baek Kim, Shugeng Cao, Joo-Won Nam, Hyukjae Choi

**Affiliations:** 1 Advanced Radiation Technology Institute, Korea Atomic Energy Research Institute, Jeongeup-si 56212, Korea; hrpark@kaeri.re.kr (H.R.P.); boram0307@hnibr.re.kr (B.-R.K.); yrkim327@kaeri.re.kr (Y.-R.K.); hhp856@kaeri.re.kr (H.H.P.); parksj94@kaeri.re.kr (J.P.); chjin@kaeri.re.kr (C.H.J.); jmkim0803@kaeri.re.kr (J.M.K.); soonjaekwon@kaeri.re.kr (S.-J.K.); jbkim74@kaeri.re.kr (J.-B.K.); 2 College of Pharmacy, Yeungnam University, Gyeongsan 38541, Korea; canta87@ynu.ac.kr (G.J.K.); jwnam@yu.ac.kr (J.-W.N.); 3 Research Institute of Cell Culture, Yeungnam University, Gyeongsan 38541, Korea; 4 Natural Product Research Division, Honam National Institute of Biological Resources, Mokpo-si 58762, Korea; 5 Daniel K. Inouye College of Pharmacy, University of Hawaii at Hilo, Hilo, HI 96720, USA; scao@hawaii.edu

**Keywords:** lentil, *Lens culinaris*, Fabaceae, LC-MS/MS, molecular networking, 18:0 Lyso PC, high-fat diet, anti-obesity

## Abstract

Lentil (*Lens culinaris*; Fabaceae), one of the major pulse crops in the world, is an important source of proteins, prebiotics, lipids, and essential minerals as well as functional components such as flavonoids, polyphenols, and phenolic acids. To improve crop nutritional and medicinal traits, hybridization and mutation are widely used in plant breeding research. In this study, mutant lentil populations were generated by γ-irradiation for the development of new cultivars by inducing genetic diversity. Molecular networking via Global Natural Product Social Molecular Networking web platform and dipeptidyl peptide-IV inhibitor screening assay were utilized as tools for structure-based discovery of active components in active mutant lines selected among the lentil population. The bioactivity-based molecular networking analysis resulted in the annotation of the molecular class of phosphatidylcholine (PC) from the most active mutant line. Among PCs, 1-stearoyl-2-hydroxy-*sn*-glycero-3-phosphocholine (18:0 Lyso PC) was selected for further in vivo study of anti-obesity effect in a high-fat diet (HFD)-induced obese mouse model. The administration of 18:0 Lyso PC not only prevented body weight gain and decreased relative gonadal adipose tissue weight, but also attenuated the levels of total cholesterol, triglycerides, low-density lipoprotein cholesterol, and leptin in the sera of HFD-induced obese mice. Additionally, 18:0 Lyso PC treatment inhibited the increase of adipocyte area and crown-like structures in adipose tissue. Therefore, these results suggest that 18:0 Lyso PC is a potential compound to have protective effects against obesity, improving obese phenotype induced by HFD.

## 1. Introduction

Lentil (*Lens culinaris*; Fabaceae) is a pulse crop cultivated and used as a foodstuff worldwide [1]. Lentils have important nutritional and health-promoting properties, and the major nutrients in lentil are carbohydrates (approximately 56%) and proteins (approximately 21%) derived from its amino acid content [2]. The essential amino acids in lentil are arginine, leucine, and lysine [3]. Bioactive peptides are also produced from lentil [4]. Lentil also contains dietary fibers, minerals, vitamins, and secondary metabolites such as phenolics, flavonoids, polyphenols, saponins, and fatty acids [5,6,7,8,9,10,11]. Lentils and its constituent have diverse biological activities such as antioxidant [5,6,9], α-glucosidase inhibitory [6], anti-inflammatory [7], and anticancer effects [8].

Numerous legumes have been bred through crossbreeding and mutagenesis to improve stress resistance, crop productivity, and nutritional traits. Mutation breeding has the advantage of improving genetic traits while maintaining superior traits in the original cultivar, by artificially inducing natural mutations [12]. Mutagenic sauces used in mutagenesis include chemical mutagens such as ethyl methane sulphonate, ethyleneimine, and 1-methyl-1-nitrosourea [13] and physical triggers such as ultraviolet lights, γ-rays, and ion beams [14]. More than 3200 officially released mutant cultivars from 214 different plant species have been registered in the Food and Agriculture Organization/International Atomic Energy Agency (FAO/IAEA) Mutant Variety Database, and most of which were developed by γ-irradiation [15]. As part of our research project for the development of functionally improved crop varieties by inducing genetic diversity through radiation breeding, our research group has developed mutant lentil populations (100 lines) by γ-irradiation of an original variety, L8.

To screen the bioactivities of L8 and the gamma-irradiated mutant lines of L8, dipeptidyl peptide (DPP)-IV inhibitor screening assay was conducted. DPP-IV is responsible for degrading and inactivating the incretins, glucose-dependent insulinotropic polypeptide and glucagon-like peptide-1; thus, DPP-IV inhibition stimulates pancreatic insulin secretion and inhibits glucagon production, leading to improved glycemic control [16]. DPP-IV levels are increased in obese and insulin-resistant subjects [17]. DPP-IV is released in a differentiation-dependent manner from adipocytes, correlating with serum insulin, leptin and adipocyte size of subcutaneous adipose tissue [18], but also shows higher release from visceral adipose tissue in associated in adipose tissue inflammation [19,20]. Thus, several studies are being explored to find DPP-IV inhibitors in natural products [21,22,23].

To identify marker metabolites that discriminate between active/inactive lentil samples, we used a feature-based molecular networking (FBMN) as an analysis method in the Global Natural Product Social Molecular Networking (GNPS) web platform for visualizing and annotating chemical composition with non-targeted mass spectrometry data of lentil samples [24]. The introduction of the GNPS web platform [25] enables automated mining of spectral data for large-scale experiments with thousands of samples in a matter of hours [26]. The molecules can be annotated from openly accessed MS/MS spectra on GNPS based on the similarity in MS/MS fragments [26,27]. In addition, MS/MS molecular networking can be used to propagate annotations to unknown metabolites [26,27]. Therefore, GNPS and molecular networking have been used to prioritize molecular weights based on observations of known and unknown analogs or distinct clusters (molecular families) of MS/MS spectra [25,26]. However, since molecular networking on GNPS is designed for MS/MS spectra comparison, it does not consider the molecular ion having the same *m/z* values. Furthermore, the concept of FBMN has emerged, which integrates MS/MS molecular networking and features of the node including retention time, and intensity of node [24]. This FBMN strategy can provide the high-throughput molecular annotation by emphasizing molecular families and differentiation of node via GNPS and molecular networking [24].

In this study, 70% acetone extracts of lentil lines were subjected to untargeted metabolite analysis using FBMN on GNPS web-platform and evaluated for their DPP-IV inhibitory activities. By the bioactivity-based molecular networking analysis, the molecular class of phosphatidylcholine (PC) that was distinguished from the most active lentil mutant line was derived. We examined the anti-obesity effect of 1-stearoyl-2-hydoxy-sn-glycero-3-phosphocholine (18:0 Lyso PC), a single compound among the PCs, by evaluating obesity-related indicators and histology in C57BL/6 male mice fed a high-fat diet (HFD).

## 2. Results

### 2.1. Molecular Networking-Based Metabolome and Dipeptidyl Peptide-IV Inhibitory Activities Analyses of Lentil Lines

**Figure 1:**
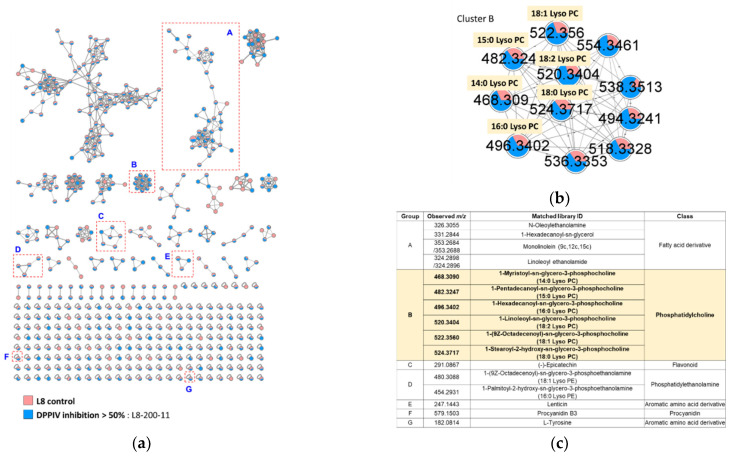
Feature-based molecular networking of lentil samples: (**a**) an entire molecular network of L8 control and L8-200-11 (most active sample); (**b**) targeted cluster annotation for the active lentil mutant lines extracts; (**c**) annotated molecular node by the comparison of Global Natural Product Social Molecular Networking library.

To employ the FBMN-based metabolomics, the 70% acetone extracts of 100 lentil mutant lines and its original line were firstly analyzed by LC-MS/MS data processed by the MZmine2 algorithms. The FBMN results allowed the annotations of 16 molecular families (Figure S1 and Table S1). These extracts were also evaluated for DPP-IV inhibitory activity at a concentration of 100 μg/mL. Among them, the extract of L8-200-11 showed DPP-IV inhibitory activity with 56.38% inhibition. The extract of lentil original line (L8) exhibited no activity with 9.44% inhibition. By combining this information, a non-targeted metabolite analysis on active/inactive lentil samples was conducted by molecular networking throughout the GNPS web platform to cluster similar spectra as molecular families (Figure 1a), and molecule annotations of each indicated cluster are presented in Figure 1c. In further analysis of FBMN applied to the most active lentil mutant line (L8-200-11) and L8 control, the active extract of L8-200-11 exhibited many spectral nodes grouped within molecular families of PC derivatives, including the cluster of B (Figure 1b). Their spectral annotations were retrieved from MS/MS spectral libraries on GNPS as following: 1-myristoyl-*sn*-glycero-3-phosphocholine (14:0 Lyso PC) at *m/z* 468.3090, 1-pentadecanoyl-*sn*-glycero-3-phosphocholine (15:0 Lyso PC) at *m*/*z* 482.3247, 1-hexadecanoyl-*sn*-glycero-3-phosphocholine (16:0 Lyso PC) at *m/z* 496.3402, 1-linoleoyl-sn-glycero-3-phosphocholine (18:2 Lyso PC) at *m/z* 520.3404, 1-(9*Z*-octadecenoyl)-*sn*-glycero-3-phosphocholine (18:1 Lyso PC) at *m/z* 522.3560, and 1-Stearoyl-2-hydroxy-*sn*-glycero-3-phosphocholine (18:0 Lyso PC) at *m/z* 524.3717.

For further in vivo study, we selected a commercially available compound, 18:0 Lyso PC contained in the most active mutant lentil (L8-200-11) extract, among PC derivatives that were annotated from the result of the FBMN-based untargeted metabolite analysis.

### 2.2. Effect of 18:0 Lyso PC on Body Weight Gonadal Adipose Tissue Weights, and Liver Weights in High-Fat Diet-Fed Mice

**Figure 2:**
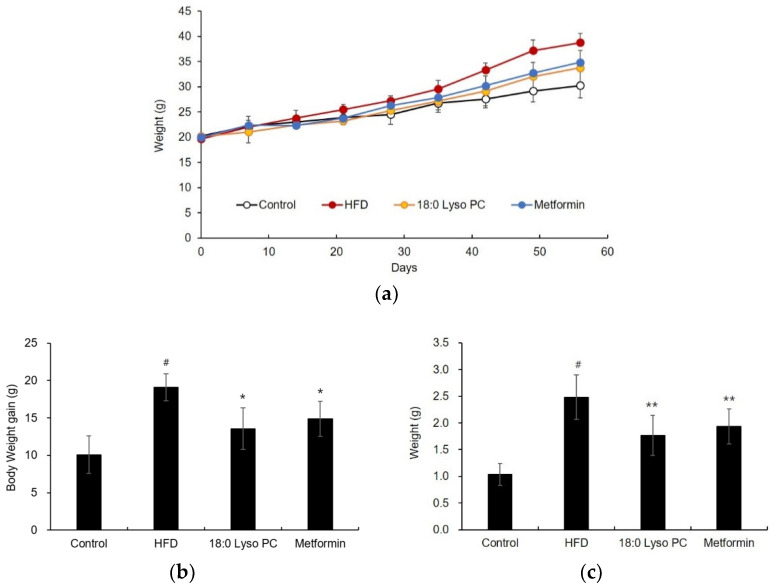
Effect of 18:0 Lyso PC on (**a**) changes in body weight during the entire experimental period (significant differences *p* values are presented in Table S2), (**b**) body weight gain and (**c**) gonadal adipose tissue weight in high-fat diet-fed mice after 8 weeks. The results are presented as mean ± SD (*n* = 6). Significant differences were identified at ^#^ *p* < 0.005, the control group vs. the high-fat diet (HFD) group; * *p* < 0.005 and ** *p* < 0.05, the HFD group vs. the treatment group.

To investigate the efficacy of 18:0 Lyso PC on HFD-induced mice model, 18:0 Lyso PC and the positive control, metformin (each 100 mg/kg/day) were administered to C57BL/6 mice fed HFD for 8 weeks. Figure 2a presents the changes in body weight during the entire experimental period. The initial body weight among the groups was not different; however, the body weight of the HFD group gradually increased compared to that of the control group. After the last treatments, the body weight gain of the control group was 10.07 ± 2.49 g, whereas the HFD group had a significantly higher body weight gain (19.09 ± 1.81 g). The 18:0 Lyso PC treatment group showed significantly reduced body weight gain due to HFD, decreasing the body weight gain by 13.56 ± 2.75 g. At the end of the experiment, gonadal adipose tissue and liver tissue were dissected and weighed. The gonadal adipose tissue weight in the control group was 1.03 ± 0.21 g, while its weight in the HFD group significantly increased to 2.48 ± 0.41 g (Figure 2c). This increase was reduced to 1.76 ± 0.37 g by the administration of 18:0 Lyso PC. In the measurement of liver tissue weight, the 18:0 Lyso PC treatment group showed a slight decrease in liver weight to 1.30 ± 0.06 g, compared to that of the HFD group (1.41 ± 0.09 g), and it was the only significant difference between the groups (Figure S2). Therefore, these results suggested that the efficacy of the 18:0 Lyso PC treatment on body weight gain and gonadal adipose tissue weight were as effective as that of treatment with the positive control, metformin.

### 2.3. Effect of 18:0 Lyso PC on Histological Analysis of Gonadal Adipose Tissue of HFD-Induced Obese Mice

**Figure 3:**
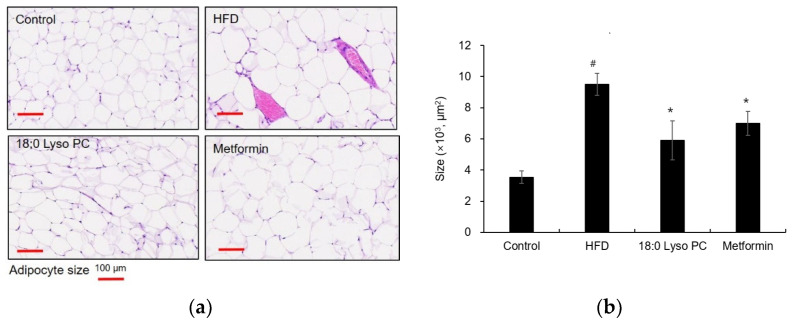
Histological analysis of gonadal adipose tissue in high-fat diet-fed mice. (**a**) Hematoxylin and eosin-stained images for adipocyte area from different groups (scale bar, 100 μm), (**b**) the distribution of adipocyte area in adipose tissue (μm^2^). The results are presented as mean ± SD (*n* = 6). Significant differences were identified at ^#^ *p* < 0.005, the control group vs. the high-fat diet (HFD) group; * *p* < 0.005, the HFD group vs. the treatment group.

Hematoxylin and eosin (H&E) staining was performed to examine the adipose size. In morphological changes in the gonadal adipocytes, the HFD group showed distinct hypertrophy compared with the control group (Figure 3a), indicating the adipocyte size of the HFD group was more than doubled compared to that of the control group (Figure 3b). The 18:0 Lyso PC treatment group demonstrated weak hypertrophy compared with the HFD diet group, suggesting that the 18:0 Lyso PC treatment significantly prevented the increase in adipocyte area due to high-fat diet.

### 2.4. Effect of 18:0 Lyso PC on Lipid Profiles in HFD-Induced Obese Mice

**Figure 4:**
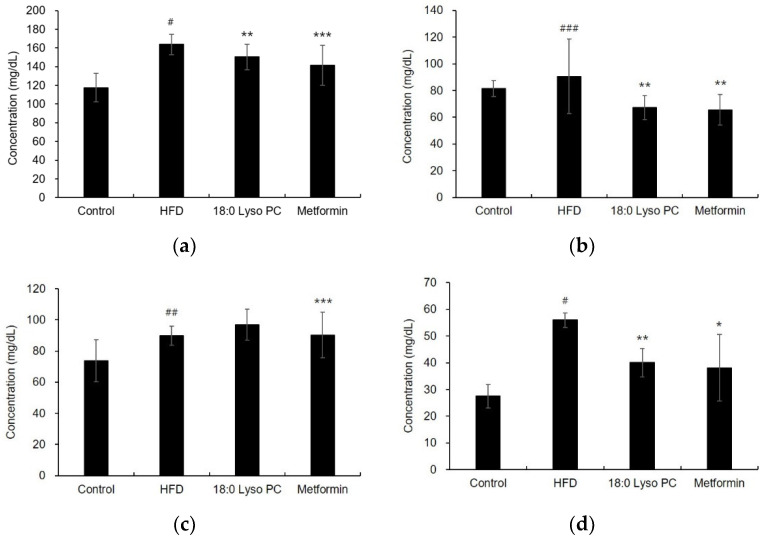
Effect of 18:0 Lyso PC on biochemical parameters in serum of high-fat diet-fed mice: the concentrations of (**a**) total cholesterol, (**b**) triglycerides, (**c**) high-density lipoprotein cholesterol, (**d**) calculated-low-density lipoprotein cholesterol. The results are presented as mean ± SD (*n* = 6). Significant differences were identified at ^#^ *p* < 0.005, ^##^ *p* < 0.05, and ^###^ *p* < 0.5, the control group vs. the high-fat diet (HFD) group; * *p* < 0.005, ** *p* < 0.05, and *** *p* < 0.5, the HFD group vs. the treatment group.

The HFD group had a significantly increased levels of total cholesterol (TC), triglycerol (TG), high-density lipoprotein (HDL) cholesterol, and calculated low-density lipoprotein (c-LDL) cholesterol in the serum compared to the control group, as shown in Figure 4a–d, The increased concentrations of TC (163.80 ± 10.94 and mg/dL) and TG (90.60 ± 27.99 mg/dL) in the HFD group were decreased to 150.50 ± 13.60 and mg/dL and 67.25 ± 8.86 mg/dL by the administration of 18:0 Lyso PC, respectively. The levels of c-LDL cholesterol were calculated using the Friedewald formula: (c-LDL = TC-HDL-(TG/5)). The HFD group had significantly elevated level of c-LDL cholesterol compared with the control group. The administration of 18:0 Lyso PC significantly reduced the c-LDL cholesterol level to 40.05 ± 5.31 mg/dL compared to that in the HFD group (55.98 ± 2.72 mg/dL). Serum lipid parameters were measured to test the effect of 18:0 Lyso PC on the improvement of hyperlipidemia in obese mice. To investigate the effect of 18:0 Lyso PC on non-alcoholic fatty liver disease, the concentrations of aspartate transaminase (AST) and alanine transaminase (ALT) in the serum were measured. The treatment of 18:0 Lyso PC decreased the elevated ALT levels in HFD-fed mice, indicating protection from liver damage; however, the protective effect was not significant (Figure S3).

### 2.5. Effect of 18:0 Lyso PC on Adipokines Levels in HFD-Induced Obese Mice

**Figure 5:**
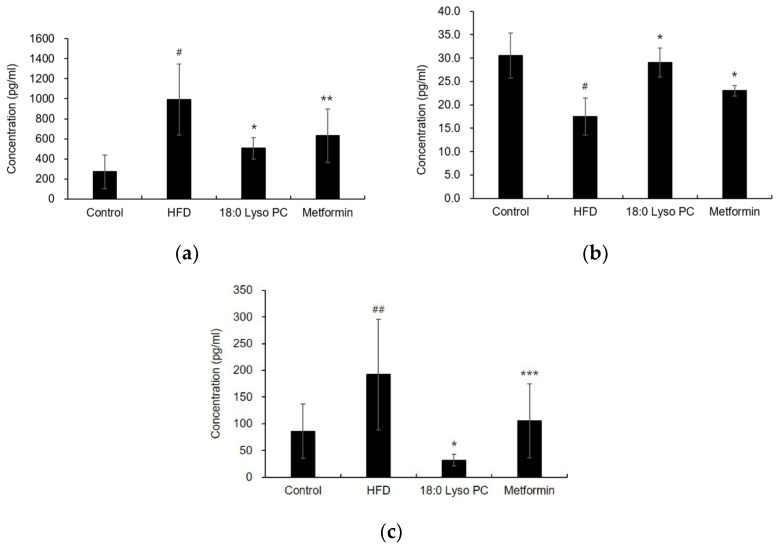
Effect of 18:0 Lyso PC on adipokines levels in serum of high-fat diet-fed mice. The concentrations of (**a**) leptin, (**b**) adiponectin, and (**c**) monocyte chemoattractant protein 1. The results are presented as mean ± SD (*n* = 6). Significant differences were identified at ^#^ *p* < 0.005 and ^##^ *p* < 0.05, the control group vs. the high-fat diet (HFD) group; * *p* < 0.005, ** *p* < 0.05, and *** *p* < 0.5, the HFD group vs. the treatment group.

Obesity increases the expression of pro-inflammatory adipokines and decreases the expression of anti-inflammatory adipokines, leading to the development of a chronic inflammatory state [28]. Leptin, one of the major adipokines produced by adipocytes, is related to pro-inflammatory cytokines and involved in body weight regulation [28,29]. The serum leptin concentration in the HFD group increased to 992.71 ± 352.41 pg/mL compared to that in the control group (271.43 ± 169.34 pg/mL); however, the administration of 18:0 Lyso PC significantly reduced the leptin level to 506.03 ± 106.98 pg/mL (Figure 5a). Meanwhile, adiponectin, an anti-inflammatory adipokine produced by adipose tissue, is inhibited by conditions associated with pro-inflammatory cytokines, hypoxia, oxidative stress, and obesity-induced adipose tissue environment [28,30,31]. When compared with the control group, the concentration of serum adiponectin in the HFD group was significantly reduced to 17.46 ± 3.96 pg/mL. The serum adiponectin levels in the 18:0 Lyso PC treatment group were significantly elevated to 29.04 ± 3.14 pg/mL, similar to the control group mice fed with a normal diet (30.56 ± 4.80 pg/mL) (Figure 5b). Monocyte chemoattractant protein 1 (MCP-1) is a type of chemokine that allows monocytes and macrophages to migrate and infiltrate the adipose tissue [32]. The expression of MCP-1 is associated with early obesity development and insulin resistance [32]. In this study, MCP-1 in the HFD group was significantly elevated to 192.22 ± 103.57 pg/mL, whereas the administration of 18:0 Lyso PC reduced MCP-1 increased by HFD to 31.77 ± 1.68 pg/mL (Figure 5c), although MCP-1 levels were lower than that in the control group (86.13 ± 50.69 pg/mL).

### 2.6. Effect of 18:0 Lyso PC on the Accumulation of Macrophages in Gonadal Adipose Tissue of HFD-Induced Obese Mice

**Figure 6:**
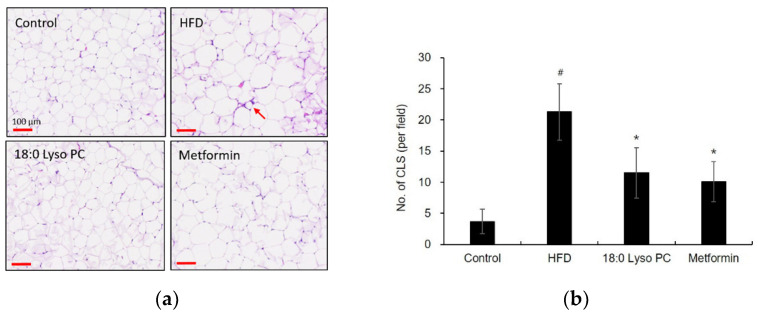
The effect of 18:0 Lyso PC on adipose tissue macrophage infiltration. (**a**) Hematoxylin and eosin (H&E) stained images for crown-like structure (CLS) from different group (scale bar, 100 μm), the arrows indicate CLS, (**b**) the quantification of CLS in adipose tissue (counts CLS per field; scale bar, 100 μm). The results are presented as mean ± SD (*n* = 6). Significant differences were identified at ^#^ *p* < 0.005, the control group vs. the high-fat diet (HFD) group; * *p* < 0.005, the HFD group vs. the treatment group.

We further confirmed whether 18:0 Lyso PC treatment could also alleviate adipose tissue macrophage infiltration. Macrophage infiltration and crown-like structure (CLS) remodeling are identified with a large number of macrophages surrounding dead adipocytes in gonadal adipose tissue of HFD-fed mice [33] (Figure S4). As shown in Figure 6a, CLSs appeared in the HFD group, but few CLSs were observed in the control group. The number of CLSs in the HFD group was about 20, but that of the 18:0 Lyso PC- or metformin-administered groups was about 10. The 18:0 Lyso PC administration group showed a significant decrease in the number of CLS (Figure 5b), suggesting that the administration of 18:0 Lyso PC might ameliorate macrophage infiltration in adipose tissue.

## 3. Discussion

An analytical workflow based on MS/MS molecular networking can tentatively annotate and classify metabolites with high-throughput process. Based on putative annotation, the molecular families of lentil original cultivar and its radiation mutant lines were characterized, analyzed, and visualized, allowing the digitization of the diversity and distribution of metabolites in lentil samples. In addition, the analytical workflow was combined with the bioactivity results to obtain to an enhanced bioactive molecular network and to presume bioactive candidate molecules [27]. This is first research on the application of molecular networking-based metabolite analysis on lentil and its mutant lines, accelerating the screening of potential mutant line for the development of new cultivar and the identification of secondary metabolites distinguished from the selected mutant line as biomarkers. From this series of analytical workflow, three lentil mutant lines with potential efficacy have been identified, but the amount of raw materials was insufficient to prepare extract samples to conduct in vivo experiments. Therefore, in this study, 18:0 Lyso PC, one of PC derivatives specifically expressed from the lentil mutant line with the greatest DPP-IV inhibitory activity was selected, and its efficacy was analyzed in the HFD-induced obese mice model.

PC is the abundant phospholipid in natural products and consists of a glycerol moiety, one or two fatty acid chains attached at the *sn*-1 and *sn*-2 positions, and a phosphorylcholine group [34]. In addition, PCs from different natural sources vary widely in their fatty acid compositions: soybean-PC is composed of unsaturated fatty acids, such as oleic acid or linoleic acid [35]; egg-PC is rich in unsaturated fatty acids [36]; and PCs from marine sources are mainly composed of eicosapentaenoic acid (EPA) and docosahexaenoic acid (DHA), which belong to the omega-3 polyunsaturated fatty acid (ω3-PUFA) group [37]. PCs alleviate obesity and obesity-related disorders in animal models [35,38,39,40]. Soybean-derived PC prevents obesity and alleviates hyperlipidemia by decreasing TG and TC [35]. HFD-induced increase in liver weight and elevated levels of AST and ALT were also decreased by the treatment of soybean-PC [35]. There has also been a report on the effects of dietary soybean PC on the prevention and treatment of hyperlipidemia and related atherosclerosis by regulation of lipid homeostasis and the interrelationships between lipoproteins [38]. ω3-PUFAs contacting PC prevent obesity-related disorders by suppressing fatty-acid synthesis, enhancing fatty-acid β-oxidation, and increasing the serum adiponectin level in Otsuka Long–Evans Tokushima fatty rats [39]. In evaluating the beneficial effects of EPA/DHA derived from marine sources in HFD-induced mice, they were found to ameliorate obesity, insulin resistance, and hyperglycemia [40]. However, the effects of 18:0 Lyso PC, as a single compound, on HFD-induced obese mice has not been reported. On analyzing obesity-related indicators in the HFD-fed mice administered with 18:0 Lyso PC, we found that the increased body weight gain and gonadal tissue weight were reduced in this group. Blood lipid parameters; TG, TC, and LDL cholesterol, were also reduced. There has been a report that soybean-derived PCs reduce liver weight and AST and ALT levels, which are indicators of liver damage in the HFD-induced obese mice [35]. In our study, hepatic tissue weight of the HFD group did not increase compared to the control group, suggesting that a slight decrease in liver tissue weight by the 18:0 Lyso PC treatment is not an improvement effect. There was also no significant difference in AST and ALT levels

Obesity is caused by excessive fat accumulation in the adipose tissue, and the increase in fat accumulation contributes to the development of a systemic, prolonged mild inflammatory state [41]. Obesity profoundly influences the secretion in the adipose tissue of pro- and anti-inflammatory cytokines. Adiponectin is the best known as a representative anti-inflammatory adipokine produced by adipose tissue [42]. Adiponectin-knockout mice exhibit showed an enhanced inflammatory response, suggesting an important role for adiponectin in inhibiting systemic and tissue inflammation [43]. In contrast, leptin is the most well-known pro-inflammatory adipokine that increases proportionally to white adipose tissue mass [43]. Leptin can enhance the production of several pro-inflammatory cytokines such as IL-5 and TNF-alpha, as well as chemokines such as MCP-1, in peripheral blood monocytes and macrophages of resident adipose tissue [42]. In our study, the 18:0 Lyso PC treatment group demonstrated regulation of adipokines by inducing a decrease in leptin and MCP-1 and an increase in adiponectin, 18:0 Lyso PC might alleviate a chronic low-inflammation due to obesity. Adipocyte hypertrophy causes local adipose tissue hypoxia [44], which results in adipose tissue macrophage infiltration [42]. Macrophage infiltration into adipose tissue is characterized by the development of CLS, which occurs when macrophages surround adipocytes [42]. MCP-1 is secreted form adipocytes and trigger macrophages [45]. In this study, the levels of MCP-1 in serum were significantly decreased with 18:0 Lyso PC administration and then CLS observed in the HFD-fed mice were diminished in 18:0 Lyso PC administration group, suggesting 18:0 Lyso PC administration might play a role in adipocyte necrosis alleviation.

In conclusion, this study indicated that LC-MS/MS-based molecular networking is a powerful tool for finding natural compounds with a specific scaffold of interest due to the increasing availability of all MS/MS data in public repositories, such as GNPS web platform increases. The molecular class of PC distinct from the active lentil mutant line was derived by the bioactivity-based molecular networking analysis, and from this molecular class, commercially available single compound, 18:0 Lyso PC, was selected for an in vivo study using HFD-induced obese mouse model. In addition, 18:0 Lyso PC treatment significantly decreased body weight gain, gonadal adipose tissue weight, and adipocyte size in HFD-induced obese mouse and significantly reduced the levels of serum TC and TG and calculated LDL cholesterol compared with the HFD group. In addition, the admiration of 18:0 Lyso PC decreased the elevated levels of leptin and MCP-1 and increased the reduced level of adiponectin in HFD-fed mice, and morphologically exhibited to ameliorate macrophage infiltration in adipose tissue. Therefore, our results suggest 18:0 Lyso PC may be a promising natural product for the prevention or treatment of obesity and related metabolic disorders.

## 4. Materials and Methods

### 4.1. General

LC-MS/MS was performed using a Thermo Scientific Q Exactive Focus Orbitrap LC-MS/MS system (Thermo Fisher Scientific, Waltham, MA, USA), equipped with a Phenomenex Kinetex C_18_ column (2.1 × 100 mm i.d., 2.6 μm; Phenomenex, Torrance, CA, USA). A [^60^Co]-irradiator (150 TBq capacity; AECL, Ottawa, ON, Canada) was used for γ-irradiation. Metformin and 1-stearoyl-2-hydoxy-*sn*-glycero-3-phosphocholine (18:0 Lyso PC) were purchased from Sigma-Aldrich (St. Louis, MO, USA). All other chemicals and solvents used in this study were of analytical grade (J. T. Baker, Phillipsburg, NJ, USA).

### 4.2. Plant Materials

The original lentil variety (L8) was propagated from seeds distributed from the Rural Development Administration (RDA)’s Genebank (RDA Genebank number IT229628) for one year in an experimental field at the Advanced Radiation Technology Institute, Korea Atomic Energy Research Institute, Jeongeup-si, Korea. After that, lentil seeds (L8) were irradiated with various doses of γ-rays (70, 100, 200, 300 Gy) using a labeled cobalt (^60^Co) source (150 TBq capacity; AECL) for 24 h at the Advanced Radiation Technology Institute, Korea Atomic Energy Research Institute. About 100 γ-irradiated mutant lines derived from the original lentil variety (L8) were sown and grown in the experimental field at the Korea Atomic Energy Research Institute under the constant conditions that were similar to the original lentil variety (L8) for two years (2016–2018). Seeds of these plant materials were randomly collected in July 2018. The voucher specimens were deposited at the Advanced Radiation Technology Institute, Korea Atomic Energy Research Institute.

### 4.3. Sample Preparation

The original lentil variety (L8) and its γ-irradiated mutant lines were ground into a powder using a Geno/grinder (SPEX, New York, NJ, USA). Each lentil powder (1 g) was extracted with 10 mL of 70% acetone using an ultrasonic bath for 60 min and was evaporated to produce the 70% acetone extract. Each dried 70% acetone extract (1 mg) was dissolved in 100 μL of methanol and centrifuged at 15,000× *g* to prepare stock solution for LC-MS/MS. Each of stock solution of sample was diluted with methanol to the final concentration of 500 μg/mL. For DPP-IV inhibitor screening, each dried 70% acetone extract (1 mg) was initially dissolved in dimethyl sulfoxide (DMSO) at a concentration of 100 mg/mL and was subsequently diluted to 500 μg/mL as the final concentration.

### 4.4. LC-MS/MS Analysis and Feature-Based Molecular Networking

The 70% acetone extracts of the original lentil variety (L8) and its γ-irradiated mutant lines were analyzed on a Thermo Scientific Q Exactive Focus Orbitrap LC-MS/MS system (Thermo Fisher Scientific Inc., Waltham, MA, USA) coupled with Thermo Fisher UltiMate HPLC system (Thermo Fisher Scientific Inc., Waltham, MA, USA). Each sample (500 μg/mL, 3 μL) was injected into a Phenomenex Kinetex C_18_ column (2.1 mm × 100 mm i.d., 2.6 μm; Phenomenex, Torrance, CA, USA). The flow rate was 0.3 mL/min using a mobile phase comprising 0.1% formic acid in water (*v/v*; solvent A) and 0.1% formic acid in acetonitrile (*v/v*; solvent B). Gradient elution was carried out as follows: 0–7.0 min, 10–45% B; 7.0–12.0 min, 45–100% B; 12.0–16.0 min, 100% B; 16.0–16.1 min, 100–10% B; 16.1–19.0 min, 10% B. The mass spectrometer was operated in positive ion mode for full scan of MS with the following parameters: spray voltage 3.5 kV; capillary temperature 320 °C; S-lens RF level 50; auxiliary gas heater temperature 350 °C; resolution 70,000; scan range *m/z* 150–2000; ACG target 1 × 10^6^. The data dependent mode for MS/MS was applied with the three most abundant peaks per cycle along with the following parameters: resolution 17,500; normalized collision energy 30 eV; AGC target 5 × 10^4^. The format of raw HRMS data were converted to an mzXML, a text-based format by using ProteoWizard 3.0.9935 [46]. The pre-processed data were applied to MZmine2 (version 2.53) and processed using feature detection and alignment with the following steps and parameters: mass detection for MS and MS/MS, noise level of 1.0 × 10^3^ and 1.0 × 10^1^; ADAP chromatogram builder, a minimum group size of scans of 5, a minimum group intensity threshold of 5.0 × 10^3^, a minimum highest intensity of 1.0 × 10^3^, and an *m/z* tolerance of 5 ppm; chromatogram deconvolution, a minimum peak height of 1.0 × 10^4^, a peak duration range of 0.05–10.00 min, a baseline level of 3.0 × 10^3^, an *m/z* range for MS/MS scan pairing of 0.025 Da, and an RT range for MS/MS scan pairing of 0.15 min; isotopes grouping, *m/z* tolerance of 5.0 ppm, a retention time tolerance of 0.05 min (absolute), a maximum charge set at 3, a representative isotope with the most intensity; feature alignment, an *m/z* tolerance of 5 ppm, a weight for *m/z* of 75, a retention time tolerance of 0.1 min (absolute), and a weight for RT of 25 [46]. The aligned peak list with MS/MS data was exported as a .mgf file and a quantitation table in csv format for FBMN on the GNPS web platform (https://gnps.ucsd.edu, access on 7 October 2021). The parameters of FBMN were set as follows: a precursor ion mass tolerance of 0.02 Da and a fragment ion mass tolerance of 0.02 Da. The nodes were connected when the cosine score was greater than 0.6, and the MS/MS spectrum shared at least three matching peaks (https://gnps.ucsd.edu/ProteoSAFe/status.jsp?task=597832d6e10b4cf3aff8df4ad06b1752, access on 7 October 2021). The result of FBMN was visualized by using Cytoscape (version 3.5.1) [47].

### 4.5. Dipetidyl Peptide(DPP)-IV Inhibitor Screening Assay

The prepared samples for the original lentil variety (L8) and its γ-irradiated mutant lines were analyzed for their DPP-IV inhibitory activities using a DPP-IV inhibitor screening assay kit (Cayman Chemical, Ann Arbor, MI, USA). The kit provides a fluorescence-based method for screening DPP-IV inhibitors and the assay procedure is described in our previous studies [48,49].

### 4.6. Animal Experiments

All animal care and experimental procedures were approved by the Ethics Committee) of the Advanced Radiation Technology Institute, Korea Atomic Energy Research Institute (Jeongeup-si, Republic of Korea; protocol code KAERI-IACUC-2021-009). Six-week-old male C57BL/6 mice weighing 19–20 g were purchased from Orient Bio, Seongnam-si, Korea. Mice were kept in the animal facility of the Advanced Radiation Technology Institute, Korea Atomic Energy Research Institute in controlled temperature and humidity conditions; 22 ± 15 °C and 55 ± 15% relative humidity. The mice were kept in a 12-h light/dark cycle (8:00–20:00) and food and drink were freely accessible. The mice were randomly divided into four groups (6 mice/group) according to their weight for an even distribution of each groups: the control group was fed a commercial normal diet (D12451; Orient Bio) containing 10% kilocalorie content of fat and administrated 0.05% Tween-20 in PBS via oral gavage; the HFD group was fed a commercial high-fat diet (D12450B; Orient Bio) containing 45% kilocalorie content of fat and administrated 0.05% Tween-20 in PBS via oral gavage; the treatment group was fed HFD and administrated 100 mg/kg of 18:0 Lyso PC dissolved in PBS via oral gavage; the positive control group was fed HFD and administrated 100 mg/kg of metformin dissolved in PBS via oral gavage. The dose of metformin was referenced to previous publications (approximately equivalent to 100–150 mg/kg body weight for mice) [50,51] and the dose of 18:0 Lyso PC was the same. The compositions of the normal diet and HFD are shown in Table 1. All groups were administered with their respective treatments 5 days per week for 8 weeks. Body weight of the mice and their food consumption were measured once per week and thrice per week, respectively, during the feeding period. At the end of the experiment, the mice were sacrificed under anesthesia by collecting blood from the heart. The blood was centrifuged to separate the plasma at 5000× *g* for 20 min at 4 °C. The liver and gonadal white fat pads were collected, weighted, and stored under −80 °C until further analysis.

### 4.7. Biochemical and Immunological Measurement of Serum Parameters

Triglyceride (TG), total cholesterol (TC), high-density lipoprotein (HDL) cholesterol, aspartate transaminase (AST/GOT), and alanine transaminase (ALT/GPT) in serum were measured with enzymatic colorimetric methods using a fully automatic chemical analyzer (Dri-Chem NK500i, Fuji, Tokyo, Japan). The levels of adiponectin, leptin, and MCP-1 in serum were measured using ELISA kits. Commercially available kits for analyzing adiponectin and leptin were purchased from R&D system (Minneapolis, MN, USA), whereas MCP-1 was analyzed using a kit from BD Biosciences (Franklin Lakes, NJ, USA).

### 4.8. Histological Analysis of Adipose Tissue

The white adipose tissue was fixed with a 10-fold volume of 10% neutral buffered formalin overnight. After fixing, the tissues were embedded in paraffin and sectioned and stained with H&E. Samples were observed under a Moticam 2300 system (Motic Instruments, Richmond, BC, Canada). We analyzed 70 adipocytes from each sample for analyzing adipocyte size.

### 4.9. Statistical Analysis

The data were expressed as the mean ± SD, and a statistical significance of differences between two groups was analyzed by using a Student’s *t* test with two-tailed test in GraphPad Prism software (Version 5.01, GraphPad Software, La Jolla, CA, USA).

## Figures and Tables

**Table 1:** The composition of a normal diet and high-fat diet.

	Normal Diet		High-Fat Diet	
Ingredient	gm	kcal	gm	kcal
Casein, 30 mesh	200	800	200	800
L-Cystine	3	12	3	12
Com starch	315	1260	72.8	291
Maltodextrin 10	35	140	100	400
Sucrose	350	1400	172.8	691
Cellulose, BW200	50	0	50	0
Soybean oil	25	225	25	225
Lard	20	180	177.5	1598
Mineral Mix S10026	10	0	10	0
Dicalcium phosphate	13	0	13	0
Calcium carbonate	5.5	0	5.5	0
Potassium citrate, H_2_O	16.5	0	16.5	0
Vitamin mix V10001	10	40	10	40
Choline bitartrate	2	0	2	0
FD&C yellow dye #5	0.05	0	0	0
FD&C yellow dye #40	0	0	0.05	0
Total	1055.05	4057	858.15	4057
Product	gm%	kcal%	gm%	kcal%
Protein	19.2	20	24	20
Carbohydrate	67.3	70	41	35
Fat	4.3	10	24	45
Total		100		100
kcal/gm	3.85		4.73	

## Data Availability

Data is contained within the article and Supplementary Material.

## Supplementary Materials

The following are available online, Table S1: Annotated molecular node by Global Natural Product Social Molecular Networking library in feature-based molecular networking applied with a L8 control and 100 radiated mutant samples, Table S2. Significant differences *p* values for the effect of 18:0 Lyso PC on changes in body weight during the entire experimental period, Figure S1. Feature-based molecular networking of L8 radiated mutants, Figure S2. Effect of 18:0 Lyso PC on liver weigh in high-fat diet-fed mice after 8 weeks. The results are presented as mean ± SD (*n* = 6). Significant difference was identified at * *p* < 0.05, the high-fat diet group vs the treatment group, Figure S3. Effect of 18:0 Lyso PC on (**a**) the concentrations of aspartate transaminase and (**b**) alanine transaminase in serum of high-fat diet-fed mice. Significant differences were not observed, Figure S4. Representative hematoxylin and eosin-stained images for normal adipocytes and crown-like structure (CLS) (scale bar, 100 μm), the arrows indicate CLS.

## Sample Availability

Not available.
