# Supplementary material for: 18:0 Lyso PC Derived by Bioactivity-Based Molecular Networking from Lentil Mutant Lines and Its Effects on High-Fat Diet-Induced Obese Mice
Source: Molecules. 2021 Dec 13;26(24):7547. doi: 10.3390/molecules26247547 (PMC8707596; doi:10.3390/molecules26247547)
Supplement: Supplementary file 1 [file molecules-26-07547-s001.zip › molecules-1490279-supplementary.pdf]

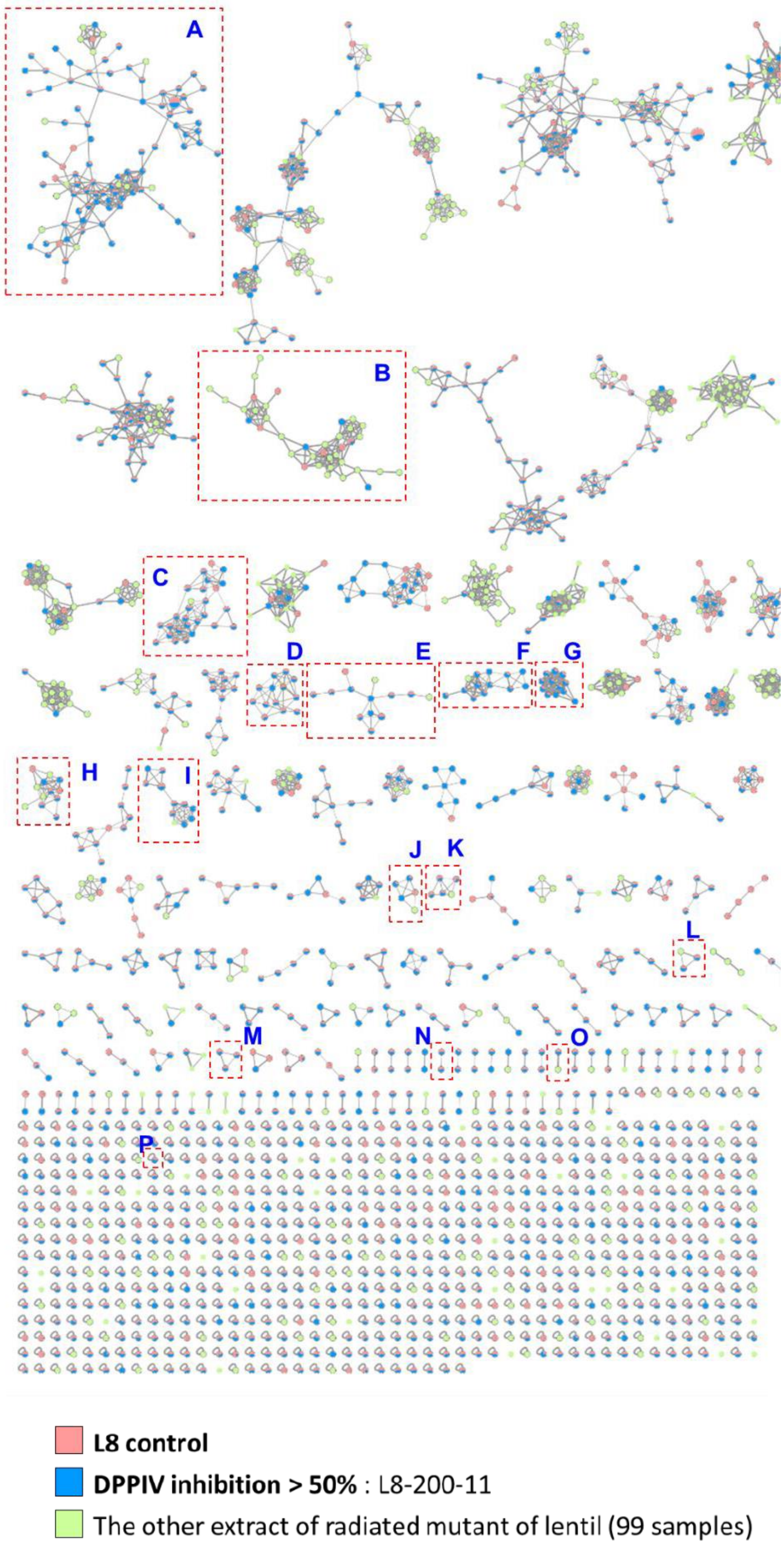

Figure S1. Feature-based molecular networking of L8 radiated mutants.

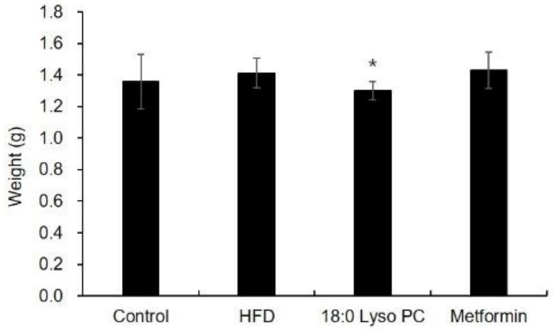

Figure S2. Effect of 18:0 Lyso PC on liver weigh in high-fat diet-fed mice after 8 weeks. The results are presented as mean ± SD (*n* = 6). Significant difference was identified at *$p < 0.05$, the high fat diet group vs the treatment group.

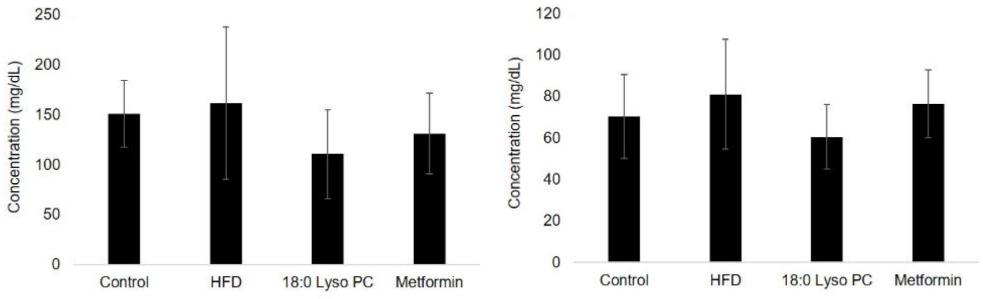

(a) (b)

Figure S3. Effect of 18:0 Lyso PC on (**a**) the concentrations of aspartate transaminase and (**b**) alanine transaminase in serum of high fat diet-fed mice. Significant differences were not observed.

Normal Diet

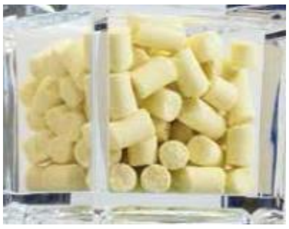

Product #	D12450B	
	gm%	*kcal%*
Protein	19.2	*20.0*
Carbohydrate	67.3	*70.0*
Fat	4.3	*10.0*
Total		*100.0*
kcal/gm	3.85	
**Ingredient**	**gm**	***kcal***
Casein, 30 Mesh	200	*800*
L-Cystine	3	*12*
Corn Starch	315	*1260*
Maltodextrin 10	35	*140*
Sucrose	350	*1400*
Cellulose, BW200	50	*0*
Soybean Oil	25	*225*
Lard	20	*180*
Mineral Mix S10026	10	*0*
DiCalcium Phosphate	13	*0*
Calcium Carbonate	5.5	*0*
Potassium Citrate, 1 H2O	16.5	*0*
Vitamin Mix V10001	10	*40*
Choline Bitartrate	2	*0*
FD&C Yellow Dye #5	0.05	*0*
FD&C Red Dye #40	0	*0*
FD&C Blue Dye #1	0	*0*
**Total**	**1055.05**	***4057***

High Fat Diet

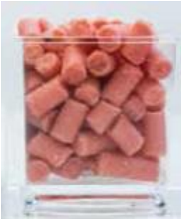

Product #	D12451	
	gm%	*kcal%*
Protein	24	*20.0*
Carbohydrate	41	*35.0*
Fat	24	*45.0*
Total		*100.0*
kcal/gm	4.73	
**Ingredient**	**gm**	***kcal***
Casein, 30 Mesh	200	*800*
L-Cystine	3	*12*
Corn Starch	72.8	*291*
Maltodextrin 10	100	*400*
Sucrose	172.8	*691*
Cellulose, BW200	50	*0*
Soybean Oil	25	*225*
Lard	177.5	*1598*
Mineral Mix S10026	10	*0*
DiCalcium Phosphate	13	*0*
Calcium Carbonate	5.5	*0*
Potassium Citrate, 1 H2O	16.5	*0*
Vitamin Mix V10001	10	*40*
Choline Bitartrate	2	*0*
FD&C Red Dye #40	0.05	*0*
FD&C Blue Dye #1	0	*0*
**Total**	**858.15**	***4057***

Figure S4. Information of the compositions of the normal diet and the high-fat diet.

Table S1. Annotated molecular node by Global Natural Product Social Molecular Networking library in feature-based molecular networking applied with a L8 control and 100 radiated mutant samples.

Group	Observed *m/z*	Matched library ID	Class
A	277.2157 /277.2161	9,12-Octadecadiynoic Acid	Fatty acid derivative
	293.2108	9(S)-HpOTrE	
	279.2318 /281.2473	Linolenic acid	
	315.2531	12,13-DiHOME	
	277.2162	9-Oxo-10E,12Z-octadecadienoic acid	
	353.2685 /353.2687 /357.2999	Monolinolenin (9c,12c,15c)	
	300.2896	Palmitoyl ethanolamide	
	324.2898 /324.2896	Linoleoyl ethanolamide	
	295.2262	13-Keto-9Z,11E-octadecadienoic acid	
	297.2424 /315.2531	9,10-Dihydroxy-12Z-octadecenoic acid	
	326.3053	N-Oleoylethanolamine	
	331.2848	1-Hexadecanoyl-sn-glycerol	
B	786.6004	1-Stearoyl-2-linoleoyl-sn-glycero-3-phosphocholine (18:0/18:2 Lyso PC)	Phosphatidylcholine
C	420.1866	Phenylmethyl-6-O-[(2R,3R,4R)-tetrahydro-3,4-dihydroxy-4-(hydroxymethyl)-2-furanyl]-beta-D-Glucopyranoside	Carbohydrate
	325.1130	4-O-beta-Galactopyranosyl-D-mannopyranose	
	360.1502	Isomaltulose	
D	291.0865	Catechin	Flavonoid
	453.1394	(2R,3R,4S,5S,6R)-2-[[2-(3,4-Dihydroxyphenyl)-5,7-dihydroxy-3,4-dihydro-2H-chromen-3-yl]oxy]-6-(hydroxymethyl)oxane-3,4,5-triol	
E	518.3212	1-Hexadecanoyl-sn-glycero-3-phosphocholine (16:0 Lyso PC)	Phosphatidylcholine
	544.3371	1-(9Z-Octadecenoyl)-sn-glycero-3-phosphocholine (18:1 Lyso PC)	
F	175.1188	Arginine	Amino acid derivative
G	518.3328	1-(9Z,12Z,15Z-octadecatrienoyl)-sn-glycero-3-phosphocholine (18:3 Lyso PC)	Phosphatidylcholine
	482.3247	1-Pentadecanoyl-sn-glycero-3-phosphocholine (15:0 Lyso PC)	
	524.3717	1-Stearoyl-2-hydroxy-sn-glycero-3-phosphocholine (18:0 Lyso PC)	
	522.3559	1-(9Z-Octadecenoyl)-sn-glycero-3-phosphocholine (18:1 Lyso PC)	
	468.3089	1-Myristoyl-sn-glycero-3-phosphocholine (14:0 Lyso PC)	
H	480.3086	1-(9Z-Octadecenoyl)-sn-glycero-3-phosphoethanolamine (18:1 Lyso PE)	Phosphatidylethanolamine
I	247.1442	Lenticin	Aromatic amino acid derivative
J	328.1392	N-(1-Deoxy-1-fructosyl)phenylalanine	Aromatic amino acid derivative
K	757.2191	5,7-Dihydroxy-2-[4-[(2S,3R,4S,5S,6R)-3,4,5-trihydroxy-6-(hydroxymethyl)oxan-2-yl]oxyphenyl]-3-[(2S,3R,4S,5S,6R)-3,4,5-trihydroxy-6-[[(2R,3R,4R,5R,6S)-3,4,5-trihydroxy-6-methyloxan-2-yl]oxymethyl]oxan-2-yl]oxychromen-4-one	Flavonoid glycoside

L	387.2015	(4S)-4-Hydroxy-3,5,5-trimethyl-4-[(E)-3-[(2R,3R,4S,5S,6R)-3,4,5-trihydroxy-6-(hydroxymethyl)oxan-2-yl]oxybut-1-enyl]cyclohex-2-en-1-one	Terpenoid glycoside
M	205.0972	L-Tryptophan	Aromatic amino acid derivative
N	182.0813	L-Tyrosine	Aromatic amino acid derivative
O	579.1501	Procyanidin B3	Procyanidin
P	255.0652	7,4′-Dihydroxyflavone	Flavonoid

Table S2. Significant differences *p* values for the effect of 18:0 Lyso PC on changes in body weight during the entire experimental period.

	Days								
	0	7	14	21	28	35	42	49	56
Control vs HFD	0.337225	0.800899	0.295365	0.006532	0.002758	0.003044	0.000005	0.000003	0.000002
HFD vs 18:0 Lyso PC	0.417169	0.389523	0.077717	0.000657	0.006785	0.039776	0.003320	0.003589	0.001395
HFD vs metformin	0.529554	0.655448	0.108034	0.003841	0.129756	0.072521	0.004325	0.002096	0.004165
